# Epidemiological, clinical, and laboratory features of patients infected with *Elizabethkingia meningoseptica* at a tertiary hospital in Hefei City, China

**DOI:** 10.3389/fpubh.2022.964046

**Published:** 2022-09-20

**Authors:** Yajuan Li, Tingting Liu, Cuixiao Shi, Bo Wang, Tingting Li, Ying Huang, Yuanhong Xu, Ling Tang

**Affiliations:** Department of Clinical Laboratory, First Affiliated Hospital of Anhui Medical University, Hefei, China

**Keywords:** *Elizabethkingia meningoseptica*, infection, clinical and laboratory features, fever, hepatic disease

## Abstract

**Background:**

*Elizabethkingia meningoseptica* is a bacterium causing potential nosocomial infections and is associated with a high mortality rate; however, the date of patients in the Hefei population who have been diagnosed with this infection is generally limited.

**Purpose:**

The clinical and laboratory data of patients from a tertiary hospital in Hefei City who had *E. meningoseptica* infection were evaluated in this retrospective analysis.

**Patients and methods:**

From May 2017 to November 2021, there were 24 patients infected with *E. meningoseptica* in the First Affiliated Hospital of Anhui Medical University. Data were gathered from the hospital's electronic medical records for all patients.

**Results:**

The most prevalent symptom among the 24 patients was fever (83.3%), followed by edema (41.7%), cough (37.5%), altered consciousness (41.7%), and sputum (37.5%), and laboratory results presented with anemia (75%), hypoproteinemia (75%), elevated C-reactive protein (CRP) (66.7%), neutrophilia (54.2%), and leukocytosis (50.0%). Hepatic disease (1 vs. 7, *P* = 0.009) was the only significant risk factor for underlying diseases. The mean value of lymphocyte (LYMPH#) (1.4 vs. 0.83 × 10^9^/L, *P* = 0.033) counts was higher in the survival group than death group, while both anemia (8 vs. 10, *P* = 0.024) and hypoproteinemia (8 vs. 10, *P* = 0.024) occurred more frequently in the death group compared with the survival one.

**Conclusion:**

Fever was the most common symptom and the only significant factor of underlying diseases was hepatic disease (*P* = 0.009) that often occurred in death groups. In this investigation, the risk factors for death in patients were anemia, hypoproteinemia, and lymphocyte count. The susceptibility of some quinolones, piperacillin-tazobactam, and cotrimoxazole was relatively high, suggesting that they may be the preferred drugs for the treatment of *E. meningoseptica* infection. As *E. meningoseptica* can produce biofilm to pollute the hospital environment and cause infection in patients, the disinfection of the hospital environment should be strengthened and medical staff should pay attention to aseptic operations.

## Introduction

*Elizabethkingia meningoseptica*, formerly known as *Chryseobacterium meningosepticum* or *Flavobacterium meningosepticum*, is a gram-negative rod that is aerobic, non-motile, non-fermenting, and does not generate spores (1). The bacteria are distributed widely in nature, including in water, fish, soils, insects, and frogs. They are also present in hospital settings, where they may contaminate medical equipment and flushing solutions (1, 2). Infections with *E. meningoseptica* are usually involved with indwelling devices and often influence immunocompromised patients, as well as neonates with neonatal meningitis and sepsis (1). It is an opportunistic pathogen that forms biofilms and can survive for extended periods in moist environments or water sources, including tap water (3). Currently, the genus *Elizabethkingia* contains six species: *Elizabethkingia anophelis, E. meningoseptica, Elizabethkingia bruuniana, Elizabethkingia miricola, Elizabethkingia occulta*, and *Elizabethkingia ursingii* (4). *E. meningoseptica* is the most virulent of the six known *Elizabethkingia* bacterial species (5). Furthermore, *E. meningoseptica* is a hospital pathogen, and correct identification of pathogens is essential for clinical diagnosis and treatment (6). Because of the intrinsic multidrug resistance of *E. meningoseptica* to commonly used antibiotics including aminoglycosides and β-lactams, the infection caused by this bacteria is difficult to cure and has a high mortality rate (1, 7). According to a study in Australia, antimicrobial resistance (AMR) genes, *bla*_*BlaB*_, *bla*_*GOB*_, and *bla*_*CME*_, were discovered in the genomes of all *Elizabethkingia* clinical isolates from Australia. Because of unique metallo-β-lactamases and unique extended-spectrum β-lactamases, *Elizabethkingia* species are considered to be resistant to most β-lactams (8).

In clinical microbiology laboratories, the matrix-assisted laser desorption/ionization time-of-flight mass spectrometry (MALDI-TOF-MS) system with an extended spectral database is extensively employed for microbial identification. It successfully identifies *E. meningoseptica* and *E. anophelis* but is unable to differentiate between the remaining species. Accurate species identification requires molecular approaches, such as whole genome sequencing and housekeeping gene sequencing (2, 9). 16S rRNA gene sequencing is regarded as an accurate approach for identifying *Elizabethkingia* species, according to the published studies (10). Some studies used sequencing of the 16S rRNA gene as a standard to verify the accuracy of the species identification system. The results of recognition of *E. meningoseptica* and *E. anophelis* were almost the same (6, 8, 10–12).

However, the clinical importance of isolating *E. meningoseptica* has always been questioned due to its poor pathogenicity. According to estimates from two medical centers in Taiwan, the annual incidence of *E. meningoseptica* infection has increased over the last decade (7). Studies have reported that almost all cases infected with *E. meningoseptica* occurred in the hospital environment, and the data on the incidence of *E. meningoseptica* infection mainly came from Taiwan. It is a new pathogen in hospitals, which is related to the high mortality in hospitals (13, 14). Notably, *E. meningoseptica* has intrinsic resistance to many antibiotics commonly used in intensive care settings, and patients in intensive care units (ICU) are increasingly at risk for *E. meningoseptica* (3, 13). With continuing advances in healthcare, clinicians are becoming increasingly aware of its clinical importance (7). Despite this, *E. meningoseptica* infection can be easily overlooked and misdiagnosed, resulting in further challenges in clinical practice with regard to timely diagnosis.

The majority of the reported cases originated in Taiwan, with only a few instances reported from Australia, India, the United States, and Europe (15). Determining whether *E. meningoseptica* is an emerging pathogenic bacterium is critical for clinical diagnosis and therapy (16). Particularly, there have only been a few studies reporting *E. meningoseptica* in Hefei City, and knowledge of how it infects and causes the disease is extremely limited. The clinical manifestations of infection with *E. meningoseptica* are diverse. Due to their inherent carbapenem resistance, the literature has paid less attention to their clinical features (17). Therefore, this study reviewed the clinical manifestations of patients infected with *E. meningoseptica* in the First Affiliated Hospital of Anhui Medical University, a tertiary hospital in Hefei, China, and further evaluated the laboratory findings, treatment history, and prognosis related to the bacteria. It is anticipated that this investigation may provide useful data for enhancing the detection and diagnosis of *E. meningoseptica* infection.

## Patients and methods

### Recruitment criteria and diagnosis of *E. meningoseptica* infection

All patients infected with *E. meningoseptica* in the First Affiliated Hospital of Anhui Medical University (Hefei, China) between May 2017 and November 2021 were included in this retrospective analysis. There were no widely accepted criteria for diagnosing *E. meningoseptica* infection. Therefore, in this investigation, a case was regarded as positive if *E. meningoseptica* was isolated from typically sterile sites, such as sputum or blood, submitted to the laboratory at the time of admission, and identified by the matrix-assisted laser desorption ionization time-of-flight mass spectrometry (MALDI-TOF MS; BioMérieux, France) in the clinical microbiology laboratory. We divided people infected with *E. meningoseptica* into survival and death groups according to previous reports from Taiwan (18). The survival group refers to the patient being cured or improved when discharged from the hospital, while the death group refers to the death of the patient while treated in the hospital. Overall, 14 patients were classified as survival group and 10 patients were classified as death group.

### Data collection

Demographic information (gender, age, and occupation), laboratory, clinical, physical examination, comorbidities, basic diseases, treatment history, complications, and in-hospital outcomes (discharge clinical status and length of hospital stay) data were obtained from the hospital's electronic medical records.

### Statistical analysis

The statistical analysis was performed using version 25.0 of SPSS. Continuous variables assuming a normal distribution were provided as the mean ± standard deviation (SD) and compared using the Student's *t*-test, whereas those without a normal distribution were provided as medians [interquartile range (IQR)] and compared using the Mann–Whitney *U*-test. For categorical variables, the data were presented as *n* (%) and compared using Fisher's exact test. At a two-tailed *P*-value of < 0.05, the results were declared statistically significant.

## Results

### Demographic and epidemiological characteristics

From May 2017 to November 2021, we reviewed 24 patients infected with *E. meningoseptica* in the First Affiliated Hospital of Anhui Medical University. Because the pathogen infection is associated with high mortality (19), we further divided the patients into survival group (14 cases) and death group (10 cases), with a mortality rate of 41.6%.

The 24 hospitalized patients were mainly from the department of intensive care medicine (9/24), and specimens were mainly sputum and blood. A total of 54.2% of patients were male. The age of the patients ranged from 6 to 74 years old, and 17 of them were over 45 years old. A total of 90% of patients in the death group were over 45 years old. Seventeen of the cases (70.8%) were infected with *E. meningoseptica* in summer and autumn. As shown in Table 1, there was no apparent statistical difference among patients in the survival group and death group, in terms of age, gender, mean hospitalization time, diagnosis season, and basic disease. The median duration from admission to discharge was 36.5 days (IQR: 19–58.8). The common underlying disease was brain disease (37.5%), hypertension (37.5%), and renal disease (33.3%). The only significant factor was hepatic disease, which was more common in the death group than in the survival group (60.0 vs. 7.1%, *p*=0.009). Invasive procedures, such as arterial or venous catheterization, tracheal intubation, lumbar cistern puncture, catheter, and mechanical ventilation, were used within 30 days prior to the isolation of *E. meningoseptica*. Of a total of 12 patients, there were seven in the survival group and five in the death group. All demographic and epidemiological characteristics are described in detail in Table 1.

**Table 1:** Demographic and basic disease analysis of patients infected with *E. meningoseptica* (*N* = 24).

**Variable**	**Total** **(*N* = 24)**	**Survival** **(*N* = 14)**	**Death** **(*N* = 10)**	***P*-value**
**Age, years**				
0–45 years	7 (29.2)	6 (42.9)	1 (10.0)	0.172
>45 years	17 (70.8)	8 (57.1)	9 (90.0)	
**Gender**				
Male	11 (45.8)	8 (57.1)	3 (30.0)	0.240
Female	13 (54.2)	6 (42.9)	7 (70.0)	
**Diagnosis season (months)**				
Spring (3–5)	5 (20.8)	4 (28.6)	1 (10.0)	0.730
Summer (6–8)	8 (33.3)	5 (35.7)	3 (30.0)	
Autumn (9–11)	9 (37.5)	4 (28.6)	5 (50.0)	
Winter (12–2)	2 (8.3)	1 (7.1)	1 (10.0)	
**Mean hospitalization time (day)**	36.5 (19–58.8)	37.6 ±19.8	34.0 (20.8–59.0)	0.725
**Basic disease**				
Brain disease	9 (37.5)	5 (35.7)	4 (40.0)	1.000
Diabetes mellitus	4 (16.7)	2 (28.6)	2 (20.0)	1.000
Malignant tumor	6 (25.0)	2 (14.3)	4 (40.0)	0.192
Renal disease	8 (33.3)	5 (35.7)	3 (30.0)	1.000
Hepatic disease	7 (29.2)	1 (7.1)	6 (60.0)	0.009
Hypertension	9 (37.5)	6 (42.9)	3 (30.0)	0.678
**Invasive device 30 days before infection**	12 (50.0)	7 (50.0)	5 (50.0)	1.000

### Clinical characteristics

The most common symptom observed in the 24 patients on presentation was fever (83.3%), followed by altered consciousness (41.7%), edema (41.7%), cough (37.5%), sputum (37.5%), abdominal distension (29.2%), chest discomfort (29.2%), chilly (25.0%), abdominal pain (20.8%), fatigue (20.8%), headache (16.6%), lethargy (12.5%), and asthma (12.5%). The mean maximal body temperature was 39.2 (SD, 0.9). Regarding complications, pneumonia was very common, which was observed in 12 of the cases (50.0%), while hydrothorax was observed in 11 cases (45.8%). However, the differences in clinical characteristics between the survival and death groups were not statistically significant (*P* > 0.05; Table 2).

**Table 2:** Clinical symptoms and complications analysis of patients infected with *E. meningoseptica* (*N* = 24).

**Variable**	**Total** **(*N* = 24)**	**Survival** **(*N* = 14)**	**Death** **(*N* = 10)**	***P*-value**
**Symptoms**				
Fever	20 (83.3)	11 (78.6)	9 (90.0)	0.615
Mean maximal body temperature (°C)	39.22 ± 0.9	39.2 ± 0.9	39.1 ± 0.9	0.560
Diarrhea	2 (8.3)	2 (14.3)	0 (00.0)	0.493
Headache	4 (16.6)	3 (21.4)	1 (10.0)	0.615
Nausea and vomiting	2 (8.3)	1 (7.1)	1 (10.0)	1.000
Abdominal distension	7 (29.2)	4 (28.6)	3 (30.0)	1.000
Abdominal pain	5 (20.8)	2 (14.3)	3 (30.0)	0.615
Cough	9 (37.5)	4 (28.6)	5 (50.0)	0.403
Sputum	9 (37.5)	4 (28.6)	5 (50.0)	0.403
Altered consciousness	10 (41.7)	4 (28.6)	6 (60.0)	0.211
Seizures	2 (8.3)	0 (00.0)	2 (20.0)	0.163
Chilly	6 (25.0)	4 (28.6)	2 (20.0)	1.000
Lethargy	3 (12.5)	1 (7.1)	2 (20.0)	0.550
Anorexia	2 (8.3)	2 (14.3)	0 (00.0)	0.493
Hemoptysis	2 (8.3)	1 (7.1)	1 (10.0)	1.000
Rash	2 (8.3)	1 (7.1)	1 (10.0)	1.000
Chest discomfort	7 (29.2)	3 (21.4)	4 (40.0)	0.393
Asthma	3 (12.5)	1 (7.1)	2 (20.0)	0.550
Fatigue	5 (20.8)	3 (21.4)	2 (20.0)	1.000
Edema	10 (41.7)	7 (50.0)	3 (30.0)	0.421
**Complications**				
Pneumonia	12 (50.0)	5 (35.7)	7 (70.0)	0.214
Gastrointestinal bleeding	2 (8.3)	1 (7.1)	1 (10.0)	1.000
Subarachnoid hemorrhage	4 (16.6)	2 (14.3)	2 (20.0)	1.000
Hydrothorax	11 (45.8)	5 (35.7)	6 (60.0)	0.408

### Laboratory findings

The laboratory data of 24 patients showed that anemia (75%) and hypoproteinemia (75%) were the most common diseases. Besides, hematological changes involved 16 cases with elevated C-reactive protein (CRP) levels (66.7%), 13 cases with neutrophilia (54.2%), and 12 cases with leukocytosis (50.0%). Twenty patients had different degrees of abnormal liver functions, with at least one of the following liver enzymes, namely alanine aminotransferase (ALT), aspartate aminotransferase (AST), alkaline phosphatase (ALP), γ-glutamyl transpeptidase (GGT), or lactate dehydrogenase (LDH) being above the normal range. Overall, the median levels of ALT, AST, ALP, GGT, and LDH were 30.5 (IQR: 17.5–74.8), 44.5 (IQR: 16.3–93.5), 81.0 (IQR: 62.0–124.0), 34.0 (IQR: 21.0–84.0), and 455.5 U/L (IQR: 249.0–873.5), respectively. The examination of hemostatic functions showed that the mean value of D-dimer (D–D) was 2.2 (IQR: 0.8–12.8) μg/ml, and it was significantly higher than the normal value (0.00–0.50 μg/ml). Moreover, the mean values of activated partial thromboplastin time (APTT) and prothrombin time (PT) were 40.5 (IQR, 37.4–49.2) and 14.5 (IQR, 13.9–16.4) s, respectively, while the mean glucose was 5.5 (IQR, 4.8–6.8) mmol/L.

Anemia occurred in eight cases (57.1%) in the survival group, as well as in 10 cases (100.0%) in the death group. There was a statistically significant difference in the incidence of Anemia between the two groups (*P* = 0.024). In these diseases, hypoproteinemia (*P* = 0.024) was also statistically significant in the two groups. In addition, lymphocyte counts (LYMPH#) (*P* = 0.033) were found to be statistically different between the two groups. In this case, the mean value of LYMPH# in the survival group was 1.4 (IQR: 1.0–1.8) × 10^9^/L, and it was higher than the mean values of 0.83 (SD, 0.6) × 10^9^/L in the death group. There was no statistically significant difference in other laboratory results between the survival group and the death group (Table 3).

**Table 3:** Laboratory results of patients infected with *E. meningoseptica* (*N* = 24).

**Variable**	**Total (*N* = 24)**	**Survival (*N* = 14)**	**Death (*N* = 10)**	***P*-value**
Anemia^a^	18 (75.0)	8 (57.1)	10 (100.0)	0.024
Hb (g/L)	105.2 ± 33.1	113.7 ± 38.0	93.3 ± 21.2	0.108
RBC counts ( ×10^12^/L)	9.5 (6.9–13.8)	11.1± 5.9	9.7± 6.4	0.565
HCT (%)	31.0 ± 9.3	33.5 ± 10.0.6	27.6 ± 6.2	0.104
Leukocytosis (>9.5 ×10^9^/L)	12 (50.0)	7 (50.0)	5 (50.0)	1.000
WBC counts ( ×10^9^/L)	9.5 (6.9–13.8)	11.1 ± 5.9	9.7 ± 6.4	0.565
Neutrophilia (>6.3 ×10^9^/L)	13 (54.2)	7 (50.0)	6 (60.0)	0.697
NEUT# ( ×10^9^/L)	7.9 (4.3–12.2)	7.4 (4.6–12.5)	8.2 ± 6.0	0.815
LYMPH# ( ×10^9^/L)	1.1 (0.6–1.6)	1.4 (1.0–1.8)	0.83 ± 0.6	0.033
MONO# ( ×10^9^/L)	0.4 (0.3–0.6)	0.4 (0.3–0.7)	0.4 ± 0.3	0.364
PLT count ( ×10^9^ platelets/L)	143.4 ± 81.5	166.5 ± 81.7	111.0 ± 73.0	0.101
PCT (ng/ml)	0.6 (0.2–6.8)	0.7 (0.2–7.1)	0.6 (0.2–10.5)	0.732
Elevated CRP (>10 mg/L)	16 (66.7)	8 (57.1)	8 (80.0)	0.388
CRP (mg/L)	9.8 ± 42.7	49.1 ± 39.1	76.2 ± 44.1	0.174
Na (mmol/L)	138.9 ± 8.4	137.1 ± 10.0	141.0 ± 5.9	0.301
K (mmol/L)	4.1 ± 0.9	4.1 ± 0.8	4.1 ±1	0.944
Hypoproteinemia^b^	18 (75.0)	8 (57.1)	10 (100.0)	0.024
TP (g/L)	56.7 (51.8–67.3)	57.5 ± 14.4	57.8 (51.0–67.8)	0.710
ALB (g/L)	32.4 ± 8.3	32.5 ± 9.4	32.2 ± 7.0	0.926
A/G	1.3 ± 0.4	1.4 ± 0.3	1.2 ± 0.4	0.444
TBIL (μmol/L)	14.8 (8.9–42.4)	13.2 (8.2–29.4)	22.2 (11.0–120.8)	0.107
ALT (U/L)	30.5 (17.5–74.8)	40.0 (18.5–76.8)	26.5 (16.3–95.3)	0.578
AST (U/L)	44.5 (16.3–93.5)	46.0 (18.5–87.8)	44.5 (12.5–140.8)	0.930
ALP (U/L)	81.0 (62.0–124.0)	82.0 (62.5–118.5)	79.0 (51.8–211.8)	0.926
GGT (U/L)	34.0 (21.0–84.0)	47.0 (28.0–88.0)	25.5 (15.3–144.0)	0.136
LDH (U/L)	455.5 (249.0–873.5)	536.0 (358.0–876.0)	262.0 (209.0–1404)	0.271
Creatinine (μmol/L)	73.5 (59.3–135.7)	76.5 (49.8–303.3)	73.5 (64.0–101.0)	0.884
Urea (mmol/L)	6.2 (4.6–11.8)	8.5 (4.2–13.8)	5.5 (4.8–8.8)	0.482
UA (μmol/L)	243.0 (189.0–308.0)	277.1 ± 101.5	217.5 (163.3–282.8)	0.203
eGFR1 (ml/(min*1.73 *m*^2^))	97.0 (61.0–119.0)	105.0 (45.5–128.0)	82.6 ± 32.4	0.402
CK (U/L)	170.5 (63.0–308.0)	171.0 (55.0–323.0)	114.0 (45.0–2731)	0.739
CKMB (U/L)	12.0 (5.8–40.8)	26.1 ± 24.9	6.0 (4.0–40.5)	0.230
APTT (s)	40.5 (37.4–49.2)	40.6 (36.3–50.5)	40.5 (38.6–47.6)	0.644
PT (s)	14.5 (13.9–16.4)	14.7 ± 1.4	15.0 (13.9–18.0)	0.291
INR	1.2 (1.1–1.3)	1.2 ± 0.1	1.2 (1.1–1.5)	0.355
D–D (μg/ml)	2.2 (0.8–12.8)	2.2 (0.7–12.9)	3.6 (0.7–14.5)	0.744
FDP (μg/ml)	6.2 (3.7–47.4)	5.4 (3.5–41.3)	11.8 (4.1–61.1)	0.477
FIB (g/L)	4.3 ± 2.2	4.8± 2.4	3.7 ± 1.0.9	0.256
TT (s)	16.7 (15.2–18.0)	17.4 (15.6–20.7)	16.0 (14.7–17.5)	0.166
Glu (mmol/L)	5.5 (4.8–6.8)	6.8 (5.7–8.4)	5.6 (4.9–8.4)	0.367

Data are expressed as number (%), $\bar{X}$ ± SD, or M (IQR). *P*-value: survival versus death. ^*a*^Anemia: male < 130 g/L and female <115 g/L, ^*b*^Hypoproteinemia: TP <60 g/L or ALB <35 g/L.

Hb, hemoglobin; RBC, red blood cell; HCT, hematocrit; WBC, white blood cell; NEUT#, neutrophil counts; LYMPH#, lymphocyte counts; MONO#, monocyte counts; PLT, platelet; PCT, procalcitonin; CRP, C-reactive protein; ALT, aspartate aminotransferase; AST, aspartate aminotransferase; ALP, Alkaline phosphatase; GGT, γ-glutamyl transpeptidase; LDH, lactate dehydrogenase; TP, Total Protein; ALB, Albumin; A/G, albumin/globulin ratio; TBIL, total bilirubin; UA, uric acid; Na, Natrium; K, Kalium; eGFR1, estimated glomerular filtration rate; CK, Creatine phosphokinase; CKMB, Creatine phosphokinase-MB; D-D, D-dimer; FDP, fibrinogen degradation products; INR, international normalized ratio; APTT, activated partial thromboplastin time; PT, prothrombin time; FIB, fibrinogen; TT, thrombin time; Glu, glucose. Hb, male (130–175) g/L and female (115–150) g/L; RBC, male (4.3–5.8) × 1,012/L and female (3.8-5.1) × 1,012/L; HCT, male (40–50)% and female (35.0–45.0)%; WBC, (3.5–9.5) × 109/L; NEUT#, (1.80–6.30) × 109/L; LYMPH#, (1.10–3.20) × 109/L; MONO#, (0.10–0.60) × 109/L; PLT, (125–350) × 109 g/L; CRP, (0.00–10.00) mg/L; PCT, (0.00–0.50) ng/mL; ALT, male (9–50) U/L and female (7–40) U/L; AST, male (15–40) U/L and female (13–35) U/L; ALP, male (45–125) U/L female (35–100) U/L, GGT, male (10–60) U/L and female (7–45) U/L; LDH, (120–250) U/L; TP, (65.0–85.0)g/L; ALB, (40.0–55.0) g/L; A/G, 1.20–2.40; TBIL, (0.0–23.0) μmol/L; Creatinine, male (57.0–97.0) μmol/L and female (41.0–73.0) μmol/L; Urea, male (3.10–8.00) mmol/L and female (2.60–7.50) mmol/L; UA, male (208–428) μmol/L and (155–357) μmol/L; Na, (135–145) mmol/L; K, (3.5–5.1) mmol/L; eGFR1, (90–100) ml/min; CK, male (55–170) U/L and female (30–135) U/L; CKMB, (0–26) U/L; APTT, (28–42) s; PT, (11–16) s; INR, 0.85–1.15; D-D, (0.00–0.50) μg/ml; FDP, (0.00–5.00) μg/ml; FIB, (2.00–4.00) g/L; TT, (14–21) s; Glu, (3.92–6.16) mmol/L.

### Drug susceptibility results

The resistance rate of *E. meningoseptica* to cefazolin was 100.00%, and the resistance rate to amikacin, aztreonam, imipenem, and tobramycin was 95.8%. In the death group, ceftazidime, amikacin, aztreonam, cefazolin, cefepime, ceftriaxone, imipenem, and tobramycin were the most resistant antibiotics (100.0%). The most active antibiotic to *E. meningoseptica* was cotrimoxazole (87.5%), followed by levofloxacin (75.0%), piperacillin/tazobactam (75.0%), and ciprofloxacin (58.3%). The intermediate resistance to gentamycin and piperacillin/tazobactam was found in 20.8% and 16.7%. There was no statistical significance in susceptibility, intermediate, and resistance between the survival group and death group. The full antibiotic susceptibility profiles of isolates are shown in Table 4.

**Table 4:** *In vitro* of drug susceptibility results of patients infected with *E. meningoseptica* (*N* = 24).

**Variable**	**Susceptible**				**Intermediate**				**Resistant**			
	**Total**	**Survival**	**Death**	***P*-value**	**Total**	**Survival**	**Death**	***P*-value**	**Total**	**Survival**	**Death**	***P*-value**
	**(*N* = 24)**	**(*n* = 14)**	**(*n* = 10)**		**(*N* = 24)**	**(*n* = 14)**	**(*n* = 10)**		**(*N* = 24)**	**(*n* = 14)**	**(*n* =10)**	
Ceftazidime	1 (4.2)	1 (7.1)	0 (0.0)	1.000	1 (4.2)	1 (7.1)	0 (0.0)	1.000	22 (91.7)	12 (85.7)	10 (100.0)	0.493
Amikacin	0 (0.0)	0 (0.0)	0 (0.0)	-	1 (4.2)	1 (7.1)	0 (0.0)	1.000	23 (95.8)	13 (92.9)	10 (100.0)	1.000
Aztreonam	1(4.2)	1 (7.1)	0 (0.0)	1.000	0 (0.0)	0 (0.0)	0 (0.0)	-	23 (95.8)	13 (92.9)	10 (100.0)	1.000
Cefazolin	0 (0.0)	0 (0.0)	0 (0.0)	-	0 (0.0)	0 (0.0)	0 (0.0)	-	24 (100.0)	14 (100.0)	10 (100.0)	1.000
Cefepime	1 (4.2)	1 (7.1)	0 (0.0)	1.000	1 (4.2)	1 (7.1)	0 (0.0)	1.000	22 (91.7)	12 (85.7)	10 (100.0)	0.493
Ceftriaxone	1 (4.2)	1 (7.1)	0 (0.0)	1.000	1 (4.2)	1 (7.1)	0 (0.0)	1.000	22 (91.7)	12 (85.7)	10 (100.0)	0.493
Ciprofloxacin	14 (58.3)	6 (42.9)	8 (80.0)	0.104	3 (12.5)	3 (21.4)	0 (0.0)	0.239	7 (29.2)	5 (35.7)	2 (20.0)	0.653
Gentamycin	1 (4.2)	0 (0.0)	1 (10.0)	0.417	5 (20.8)	2 (14.3)	3 (30.0)	0.615	18 (75.0)	12 (85.7)	6 (60.0)	0.192
Imipenem	1 (4.2)	1 (7.1)	0 (0.0)	1.000	0 (0.0)	0 (0.0)	0 (0.0)	-	23 (95.8)	13 (92.9)	10 (100.0)	1.000
Levofloxacin	18 (75.0)	11 (78.6)	7 (70.0)	0.665	1 (4.2)	0 (0.0)	1 (10.0)	0.417	5 (20.8)	3 (21.4)	2 (20.0)	1.000
Piperacillin/tazobactam	18 (75.0)	12 (85.7)	6 (60.0)	0.192	4 (16.7)	1 (7.1)	3 (30.0)	0.272	2 (8.3)	1 (7.1)	1 (10.0)	1.000
Tobramycin	1 (4.2)	1 (7.1)	0 (0.0)	1.000	0 (0.0)	0 (0.0)	0 (0.0)	-	23 (95.8)	13 (92.9)	10 (100.0)	1.000
Cotrimoxazole	21 (87.5)	13 (92.9)	8 (80.0)	0.550	0 (0.0)	0 (0.0)	0 (0.0)	-	3 (12.5)	1 (7.1)	2 (20.0)	0.550

## Discussion

*Elizabethkingia meningoseptica* is most commonly isolated from freshwater, saltwater, and soil, as well as from moist and dry clinical environments, intravenous lipid solutions, equipment surfaces, and municipal water supplies, including those that are adequately chlorinated. It is an opportunistic pathogen, despite being nearly widespread. *E. meningoseptica* primarily infects elderly and immunocompromised patients in intensive care settings (20). Elizabeth O. King, a bacteriologist at the Centers for Disease Control in Atlanta, Georgia, United States, was the first person to report the bacteria in 1959 (21). Infections have been reported all over the world, including in the Central African Republic, Mauritius, Singapore, Taiwan, and the United States (8). According to a study conducted in Taiwan, China, *E. meningoseptica* was the third most frequent respiratory pathogen, next to *Pseudomonas aeruginosa* and the *Acinetobacter calcoaceticus–Acinetobacter baumannii* (ACB) complex, in a medical Center in southern Taiwan. It was also the fourth most frequent pathogen of carbapenem-resistant bacteremia there (17). With the increasing incidence of *E. meningoseptica* infections, the importance of being a hospital infection bacterium is increasingly recognized by human beings. However, information regarding this disease's incidence and treatment in the city of Hefei was insufficient. Thus, clinical data for this study was retrospectively collected from the hospital's electronic medical records of 24 patients who had *E. meningoseptica* infections. In addition, demographic information, clinical and laboratory features, treatment history, and infection outcomes were reported.

Among the 24 *E. meningoseptica* clinical isolates collected from the First Affiliated Hospital of Anhui Medical University, the major sources were sputum (33.3%) and blood (25.0%), respectively. Due to the high mortality rate, we divided the patients into survival group and death group. In this retrospective analysis, we found that patients over 45 had a mortality rate of 90% compared to those under 45 who had a mortality rate of 10%. Mortality rates varied from 23% to 52% in different studies (22). In our study, 41.6% of patients died, and most of the deaths were elderly patients with impaired immune function. Infections with *E. meningoseptica* throughout the year were most prevalent in summer and autumn (70.8%). In previous studies, neonatal patients, especially premature infants, had a high risk of *E. meningoseptica* infection. In this investigation, it was discovered that all isolates, except for one, were from adults, demonstrating the low rate of infectivity rate of this bacterium in children in the province of Anhui (18, 23). Univariate analysis of a prior study revealed that individuals with liver cirrhosis (*P* = 0.032) had a higher mortality rate (4). The only risk factor for underlying diseases in this study was hepatic disease (*P* = 0.009). According to our data, hypertension (37.5%) and brain disease (37.5%) were the most prevalent underlying diseases. However, a prior study revealed that diabetes mellitus (25%) and malignancy (36%) were the most prevalent underlying diseases (18). A study found that congestive heart failure (42.1%), chronic lung disease (63.2%), and hypertension (57.9%) were the most prevalent underlying diseases (24). These inconsistent results may be attributable to geographical disparities, variations in the size of the study sample, and changes in the study design. Noteworthy, invasive catheters, such as intravascular catheters or endotracheal intubation, are the significant factors in *E. meningoseptica-*related infections (25). The outbreak of *E. meningoseptica* infection was previously reported to be related to environmental pollution. The sources of environmental pollution included contaminated syringes in refrigerators, respiratory equipment, bottles, sink taps, sink drains, tube feeding, arterial catheter flushing fluid, pressure sensors, and disinfectants. However, it has also been reported that inadequate disinfection of nipple storage tanks in baby nurseries led to infant infections (26).

Few investigations have characterized the clinical manifestations of *E. meningoseptica* infection. Fever (83.3%) was the most prevalent clinical symptom in Hefei City. It was comparable to the 83.8% fever rate recorded by a Taiwan medical Center previously (18). Most patients presented with fever, suggesting infection. Bacterial infection often causes fever, and the clinical microbial culture and identification are needed to distinguish the infections of *E. meningoseptica* and other bacteria (21, 27). Lack of fever may delay the detection of infection or just indicate a poor general state (18). According to a study conducted in Taiwan, individuals with *E. meningoseptic* bacteremia had a greater prevalence of primary bacteremia, used fewer antibiotics, and experienced less shock when the condition first manifested than patients who did not have the infection (24). The clinical symptoms of the two groups in this investigation were not significantly different. Pneumonia, sepsis, and meningitis are the three main types of infections caused by *E. meningoseptica*, but there are rare reports of abdominal infection, endocarditis, eye infection, osteomyelitis, keratitis, urinary tract infection, septic arthritis, and skin or soft tissue infection (28–30). Among the complications in this study, there were 12 cases of pneumonia (50.0%) and 11 cases of hydrothorax (45.8%). Pneumonia was a major complication in patients infected by *E. meningoseptica*. There were 70% patients with pneumonia in the death group, as well as 35.7% patients with pneumonia in the survival group.

There was very little information available in the literature about the laboratory characteristics of *E. meningoseptica*, and most previous research only included a limited number of patients. Hypoproteinemia was identified as a risk factor for mortality in patients with *E. meningoseptica* infection in a prior investigation (22). In univariate analysis, lymphocyte count (*P* = 0.033), anemia (*P* = 0.024), and hypoproteinemia (*P* = 0.024) were the risk factors for death in our study. Specifically, anemia (75%) and hypoproteinemia (75%) were the most significant clinical findings in our investigation, as they were observed in the majority of the patients. As a result, we postulated that measuring albumin (ALB), total protein (TP), and hemoglobin (Hb) levels could aid in the diagnosis of *E. meningoseptica* infection. Comparable to usual bacterial infections, peripheral blood WBC and neutrophils counts, as well as the biochemical index CRP rose in the majority of *E. meningoseptica*-infected patients. A study by Arbune et al. (31) showed an infant's laboratory results were leukocytosis with neutrophilia, and anemia. At least one increased liver enzyme, including AST, ALT, ALP, LDH, and GGT, was observed in 24 patients. Consequently, the effect of *E. meningoseptica* infection on liver function was also worthy of consideration during the progression of the disease.

The Clinical and Laboratory Standards Institute had not developed interpretive breakpoints for *E. meningoseptica* minimum inhibitory concentrations (MICs) until 2013 (13). Antibiotics, as the main factors influencing the prognosis, are an essential component of the treatment. *Elizabethkingia* species infections are difficult to cure and have a high case-fatality rate, most likely as a result of their intrinsic antibiotic resistance (32). The bacteria are susceptible to ciprofloxacin, rifampicin, trimethoprim-sulfamethoxazole, and vancomycin, but resistant to β-lactam agents, aminoglycosides, and chloramphenicol (31, 33). In a case report from Sichuan, four isolates were isolated from urine, which were resistant to aminoglycosides (amikacin, tobramycin, and gentamicin), cephalosporins (ceftazidime, cefotaxime), cefoxitin, and aztreonam, and susceptible to trimethoprim-sulfamethoxazole (30). Our observations were consistent with previous findings. In this investigation, all isolates showed resistance to cefazolin, and the majority exhibited resistance to amikacin, aztreonam, imipenem, tobramycin, ceftazidime, cefepime, ceftriaxone, and gentamycin. According to earlier research conducted in Taiwan, the isolates of *E. meningoseptica* were more susceptible to the antibiotics piperacillin (15%), levofloxacin (30%), and minocycline (60%) (4). Our study showed that the most active antibiotic was cotrimoxazole (87.5%), followed by levofloxacin (75.0%), piperacillin/tazobactam (75.0%), and ciprofloxacin (58.3%). In a hospital in Beijing, 15 of 26 *E. meningoseptica* strains were resistant to sulfamethoxazole/ trimethoprim. A polymerase chain reaction was utilized to detect the resistance-determining genes for trimethoprim/sulfamethoxazole. Six isolates held the *sul*I gene and four isolates possessed the *sul*II gene; however, just one isolate contained the *dfr*A12 gene (34). In the investigation conducted by Jian et al., all *E. meningoseptica* isolates were resistant to ciprofloxacin, whereas 44% of them were resistant to levofloxacin. This resistance was mostly mediated by a single nucleotide mutation in the *gyr*A gene QRDR (35). The difference in drug susceptibility in different studies may be due to the regional drug resistance distribution and the size of the sample base. Chiu et al. (23) reported that vancomycin was successfully used for the treatment of *E. meningoseptica* infection. And genes related to tetracycline and vancomycin resistance were detected (36). However, in other studies, vancomycin was not suggested to be effective against this microorganism (37). As this was a retrospective analysis, data on vancomycin and minocycline were not available. An important aspect of infection management is the use of antibiotics in a timely manner. In a study, there was a contradiction between the genotype and phenotype of tetracyclines, sulfonamides, and quinolones. There were no quinolone resistance genes found, and 90% of the bacteria were ciprofloxacin-resistant (38). Nevertheless, in our study, 58.3% of strains were susceptible to ciprofloxacin. Twenty-one strains of *Elizabethkingia* isolated from five hospitals in Hong Kong were susceptible to ciprofloxacin, vancomycin, and cefoperazone-sulbactam (1). The main finding of Chen et al. (39) was that the microbial cure rate of piperacillin/tazobactam combined with methoxyprolin/sulfasalazine or fluoroquinolone seemed to be high, and the mortality rate was relatively low. Fifty-one percent of 100 *E. meningoseptica* isolates in a hospital in Shanghai had moderate antibacterial activity against sitafloxacin, suggesting that sitafloxacin may be a promising treatment for *E. meningoseptica* infection (40). In terms of our drug susceptibility results, the medication to consider first should be cotrimoxazole, followed by quinolones and piperacillin/tazobactam.

It is difficult to correctly identify *E. meningoseptica* by traditional microbiological methods, and it is common to misidentify *E. anophelis* and *E. meningoseptica* (6). However, delays in biological identification may lead to treatment failure. Different AST methods (*viz*., disc diffusion, broth-micro dilution, and E-test) have different drug susceptibility results, which further complicate the treatment. Literature shows that the paper diffusion method is not reliable, the broth-micro dilution method is the preferred method. While the automatic drug susceptibility testing system is more commonly used in clinical laboratories, there are still differences between the two (11, 41, 42). As a pathogen causing nosocomial infections, there is little information about its genome composition and related characteristics in the literature. A document indicated that the genomes of *E. meningoseptica* isolated from patients carried three β-lactamases. These strains were resistant to β-lactams and cephalosporins, which may be attributed to *bla*_*CME*_; resistance to carbapenems and penicillin–β-lactamase inhibitor combinations may be attributed to *bla*_*BlaB*_ and *bla*_*GOB*_, which explains the molecular mechanism of drug resistance (8). A study in Hainan showed that clinical strains of *E. meningoseptica* had β-lactam, macrolide, tetracycline, quinolone, glycopeptide, and multidrug-resistance efflux pump genes. The clinical isolates contained antibiotic efflux pump genes, *cme*B, *ade*F, and *van*B, and glycopeptide resistance gene, *van*W, in which *van*B glycopeptide resistance gene *van*W mutation was observed to be involved in the regulation of teicoplanin resistance (38). A literature in Wuhan showed that 23 and 32 new *Bla*_*BlaB*_ and *Bla*_*GOB*_ variants were found in *Elizabethkingia* spp., respectively. Some variations did not aggregate according to the species-specific branching, indicating that the MBL gene may be transmitted across species in *Elizabethkingia* species (43). In the study of Girdhar et al. (44), they identified 18 unique proteins related to the metabolic pathway of *E. meningoseptica* from 3,391 annotated proteins, which may be the starting point for drug design and development.

In addition, we used the MALDI-TOF MS database software and constructed the development tree for homology analysis of eight strains of the total 24 *E. meningoseptica*. The tree diagram based on correlation obtained by MALDI-TOF MS clustered the eight strains in the same group, showing that they had a strong genetic relationship. We found that three strains were highly similar in *E. meningoseptica* (~87%), suggesting common hospital sources. Eight strains were identified as *E. meningoseptica* by 16S rRNA gene sequencing, which was the same as the result of MALDI-TOF MS. Since there are few cases of patients collected at present and *E. meningoseptica* that have not accumulated large data, we will continue to collect cases for analysis in the later stage to provide help for clinical diagnosis and identification.

Various studies have shown that mortality is associated with inappropriate empirical antimicrobial therapy (4, 7, 15, 18). In addition, the tendency to form biofilms poses further challenges to the treatment of patients, especially when organisms have been established on indwelling devices, such as endotracheal tubes (7). In a study from Singapore, the identification of the gene for capsule biosynthesis, *capD*, and the gene for the AdeFGH efflux pump, *adeG*, in all *Elizabethkingia* species leads to the possibility of biofilm formation, which gives the bacteria the ability to remain on varied surfaces (6). The potential of *E. meningoseptica* to produce biofilm indicates that it has the ability to impede treatment. Numerous investigations involving *E. meningoseptica* infection or outbreaks caused by flume or faucet drainage pollution have demonstrated that once biofilm is developed, it is difficult to eliminate from the environment (5, 20, 45).

This study has a number of limitations. Because this is a retrospective single-center study, some data may be lost, incomplete, or improperly reported. Furthermore, because of the study's limited sample size, the explanation for our results may not be applicable in larger populations. Despite these limitations, this work offers an important potential for the investigation of *E. meningoseptica* infection in Hefei. It is also expected that this research would offer guidance and reference for clinicians to timely diagnose and treat *E. meningoseptica* infection.

## Conclusion

In summary, the most common symptom in *E. meningoseptica* patients was fever, which was followed by altered consciousness, edema, cough, sputum, abdominal distension, chest discomfort, and chilly. The only significant factor of underlying diseases was that hepatic disease (*P* = 0.009) often occurred in death groups. A majority of patients experienced anemia and hypoproteinemia in the laboratory result. Our findings suggest that hypoproteinemia, unexplained fever, anemia, and lymphocyte count should be considered as *E. meningoseptica* selected diagnosis references for early treatment intervention. The susceptibility of some quinolones, piperacillin-tazobactam and cotrimoxazole was relatively high, suggesting that they may be the preferred drugs for the treatment of *E. meningoseptica* infection. Since such bacteria can form biofilms in a humid environment and spread with water or equipment relating to water as a source of transmission, it is necessary to strengthen the hand hygiene compliance of medical staff and control nosocomial infections to avoid the spread and spread of *E. meningoseptica* infection. This study analyzed 5 years of patient data from Anhui Province and could be used to improve strategies for preventing, diagnosing, and treating *E. meningoseptica* infections in other provinces and cities.

## Data availability statement

The original contributions presented in the study are included in the article/Supplementary material, further inquiries can be directed to the corresponding authors.

## Ethics statement

This study was reviewed and approved by the Ethics Committee of the First Affiliated Hospital of Anhui Medical University in accordance with the Declaration of Helsinki (ethical approval number: Quick-PJ 2022-10-31). Written informed consent to participate in this study was provided by the participants' legal guardian/next of kin. Written informed consent was obtained from the individual(s), and minor(s)' legal guardian/next of kin, for the publication of any potentially identifiable images or data included in this article.

## Author contributions

YL and TLiu analyzed data and wrote original draft. CS, BW, TLi, YH, YX, and LT reviewed and edited the manuscript. YX and YL provided funding. All authors have read and agreed to publish the final manuscript.

## Funding

This work was financially supported by Anhui Natural Science Foundation (grant number: 9021138201) and Scientific Research Project of Universities in Anhui Province (grant number: KJ2020A0170).

## Conflict of interest

The authors declare that the research was conducted in the absence of any commercial or financial relationships that could be construed as a potential conflict of interest.

## Publisher's note

All claims expressed in this article are solely those of the authors and do not necessarily represent those of their affiliated organizations, or those of the publisher, the editors and the reviewers. Any product that may be evaluated in this article, or claim that may be made by its manufacturer, is not guaranteed or endorsed by the publisher.
