# Corrigendum: Epidemiological, clinical, and laboratory features of patients infected with *Elizabethkingia meningoseptica* at a tertiary hospital in Hefei City, China

**DOI:** 10.3389/fpubh.2022.1054878

**Published:** 2022-11-04

**Authors:** Yajuan Li, Tingting Liu, Cuixiao Shi, Bo Wang, Tingting Li, Ying Huang, Yuanhong Xu, Ling Tang

**Affiliations:** Department of Clinical Laboratory, First Affiliated Hospital of Anhui Medical University, Hefei, China

**Keywords:** *Elizabethkingia meningoseptica*, infection, clinical and laboratory features, fever, hepatic disease

In the published article, there was an error in Table 4 “*In vitro* of drug susceptibility results of patients infected with *E. meningoseptica* (*N* = 24)” as published. We are sorry for the percentage error, which is due to format conversion in the manuscript modification. This does not influence the *P*-value and the conclusion of the article. In the survival group, 85.7% of 14 strains was susceptible to piperacillin/tazobactam and 92.9% of 14 strains was susceptible to cotrimoxazole. In the death group, 70% of 10 strains was susceptible to levofloxacin and 60% of 10 strains was susceptible to piperacillin/tazobactam. The intermediate resistance of strains in the death group to levofloxacin was 10%. The resistance rates of all strains to levofloxacin and piperacillin/tazobactam were 20.8 and 8.3%, respectively. The resistance rate of strains in the survival group to amikacin, aztreonam, imipenem, and tobramycin was 92.9%. The resistance rate of strains in the death group to levofloxacin was 20%. The corrected Table 4 and its caption appear below.

**Table 4:** The original “TABLE 4 *In vitro* of drug susceptibility results of patients infected with *E. meningoseptica* (*N* = 24).”

**Variable**	**Susceptible**				**Intermediate**				**Resistant**			
	**Total**	**Survival**	**Death**	***P*-value**	**Total**	**Survival**	**Death**	***P*-value**	**Total**	**Survival**	**Death**	***P*-value**
	**(*N* = 24)**	**(*n* = 14)**	**(*n* = 10)**		**(*N* = 24)**	**(*n* = 14)**	**(*n* = 10)**		**(*N* = 24)**	**(*n* = 14)**	**(*n* = 10)**	
Ceftazidime	1 (4.2)	1 (7.1)	0 (0.0)	1.000	1 (4.2)	1 (7.1)	0 (0.0)	1.000	22 (91.7)	12 (85.7)	10 (100.0)	0.493
Amikacin	0 (0.0)	0 (0.0)	0 (0.0)	-	1 (4.2)	1 (7.1)	0 (0.0)	1.000	23 (95.8)	13 (93.9)	10 (100.0)	1.000
Aztreonam	1(4.2)	1 (7.1)	0 (0.0)	1.000	0 (0.0)	0 (0.0)	0 (0.0)	-	23 (95.8)	13 (93.9)	10 (100.0)	1.000
Cefazolin	0 (0.0)	0 (0.0)	0 (0.0)	-	0 (0.0)	0 (0.0)	0 (0.0)	-	24 (100.0)	14 (100.0)	10 (100.0)	1.000
Cefepime	1 (4.2)	1 (7.1)	0 (0.0)	1.000	1 (4.2)	1 (7.1)	0 (0.0)	1.000	22 (91.7)	12 (85.7)	10 (100.0)	0.493
Ceftriaxone	1 (4.2)	1 (7.1)	0 (0.0)	1.000	1 (4.2)	1 (7.1)	0 (0.0)	1.000	22 (91.7)	12 (85.7)	10 (100.0)	0.493
Ciprofloxacin	14 (58.3)	6 (42.9)	8 (80.0)	0.104	3 (12.5)	3 (21.4)	0 (0.0)	0.239	7 (29.2)	5 (35.7)	2 (20.0)	0.653
Gentamycin	1 (4.2)	0 (0.0)	1 (10.0)	0.417	5 (20.8)	2 (14.3)	3 (30.0)	0.615	18 (75.0)	12 (85.7)	6 (60.0)	0.192
Imipenem	1 (4.2)	1 (7.1)	0 (0.0)	1.000	0 (0.0)	0 (0.0)	0 (0.0)	-	23 (95.8)	13 (93.9)	10 (100.0)	1.000
Levofloxacin	18 (75.0)	11 (78.6)	7 (77.8)	0.665	1 (4.2)	0 (0.0)	1 (11.1)	0.417	5 (16.6)	3 (21.4)	2 (22.2)	1.000
Piperacillin/tazobactam	18 (75.0)	12 (92.3)	6 (66.7)	0.192	4 (16.7)	1 (7.1)	3 (30.0)	0.272	2 (8.4)	1 (7.1)	1 (10.0)	1.000
Tobramycin	1 (4.2)	1 (7.1)	0 (0.0)	1.000	0 (0.0)	0 (0.0)	0 (0.0)	-	23 (95.8)	13 (93.9)	10 (100.0)	1.000
Cotrimoxazole	21 (87.5)	13 (93.9)	8 (80.0)	0.550	0 (0.0)	0 (0.0)	0 (0.0)	-	3 (12.5)	1 (7.1)	2 (20.0)	0.550

**Table 4:** The corrected “TABLE 4 *In vitro* of drug susceptibility results of patients infected with *E. meningoseptica* (*N* = 24).”

**Variable**	**Susceptible**				**Intermediate**				**Resistant**			
	**Total**	**Survival**	**Death**	***P*-value**	**Total**	**Survival**	**Death**	***P*-value**	**Total**	**Survival**	**Death**	***P*-value**
	**(*N* = 24)**	**(*n* = 14)**	**(*n* = 10)**		**(*N* = 24)**	**(*n* = 14)**	**(*n* = 10)**		**(*N* = 24)**	**(*n* = 14)**	**(*n* =10)**	
Ceftazidime	1 (4.2)	1 (7.1)	0 (0.0)	1.000	1 (4.2)	1 (7.1)	0 (0.0)	1.000	22 (91.7)	12 (85.7)	10 (100.0)	0.493
Amikacin	0 (0.0)	0 (0.0)	0 (0.0)	-	1 (4.2)	1 (7.1)	0 (0.0)	1.000	23 (95.8)	13 (92.9)	10 (100.0)	1.000
Aztreonam	1(4.2)	1 (7.1)	0 (0.0)	1.000	0 (0.0)	0 (0.0)	0 (0.0)	-	23 (95.8)	13 (92.9)	10 (100.0)	1.000
Cefazolin	0 (0.0)	0 (0.0)	0 (0.0)	-	0 (0.0)	0 (0.0)	0 (0.0)	-	24 (100.0)	14 (100.0)	10 (100.0)	1.000
Cefepime	1 (4.2)	1 (7.1)	0 (0.0)	1.000	1 (4.2)	1 (7.1)	0 (0.0)	1.000	22 (91.7)	12 (85.7)	10 (100.0)	0.493
Ceftriaxone	1 (4.2)	1 (7.1)	0 (0.0)	1.000	1 (4.2)	1 (7.1)	0 (0.0)	1.000	22 (91.7)	12 (85.7)	10 (100.0)	0.493
Ciprofloxacin	14 (58.3)	6 (42.9)	8 (80.0)	0.104	3 (12.5)	3 (21.4)	0 (0.0)	0.239	7 (29.2)	5 (35.7)	2 (20.0)	0.653
Gentamycin	1 (4.2)	0 (0.0)	1 (10.0)	0.417	5 (20.8)	2 (14.3)	3 (30.0)	0.615	18 (75.0)	12 (85.7)	6 (60.0)	0.192
Imipenem	1 (4.2)	1 (7.1)	0 (0.0)	1.000	0 (0.0)	0 (0.0)	0 (0.0)	-	23 (95.8)	13 (92.9)	10 (100.0)	1.000
Levofloxacin	18 (75.0)	11 (78.6)	7 (70.0)	0.665	1 (4.2)	0 (0.0)	1 (10.0)	0.417	5 (20.8)	3 (21.4)	2 (20.0)	1.000
Piperacillin/tazobactam	18 (75.0)	12 (85.7)	6 (60.0)	0.192	4 (16.7)	1 (7.1)	3 (30.0)	0.272	2 (8.3)	1 (7.1)	1 (10.0)	1.000
Tobramycin	1 (4.2)	1 (7.1)	0 (0.0)	1.000	0 (0.0)	0 (0.0)	0 (0.0)	-	23 (95.8)	13 (92.9)	10 (100.0)	1.000
Cotrimoxazole	21 (87.5)	13 (92.9)	8 (80.0)	0.550	0 (0.0)	0 (0.0)	0 (0.0)	-	3 (12.5)	1 (7.1)	2 (20.0)	0.550

The authors apologize for this error and state that this does not change the scientific conclusions of the article in any way. The original article has been updated.

## Publisher's note

All claims expressed in this article are solely those of the authors and do not necessarily represent those of their affiliated organizations, or those of the publisher, the editors and the reviewers. Any product that may be evaluated in this article, or claim that may be made by its manufacturer, is not guaranteed or endorsed by the publisher.

