# Supplementary material for: Epidemiological, clinical, and laboratory features of patients infected with Elizabethkingia meningoseptica at a tertiary hospital in Hefei City, China
Source: Front Public Health. 2022 Sep 20;10:964046. doi: 10.3389/fpubh.2022.964046 (PMC9549487; doi:10.3389/fpubh.2022.964046)
Supplement: Supplementary file 1 [file Data_Sheet_1.PDF]

[Download] [GenBank] [Graphics]

Elizabethkingia meningoseptica strain ATCC 13253 16S ribosomal RNA, partial sequence

Sequence ID: NR_042267.1 **Length:** 1450 **Number of Matches:** 1

Range 1: 40 to 1421 GenBank Graphics

▼ Next Match ▲ Previous Match

Score	Expect	Identities	Gaps	Strand
2453 bits(1328)	0.0	1367/1385(99%)	6/1385(0%)	Plus/Plus

```
Query  1    GCCGAGCGGT--AGATTACTTTCGGGTAA-TTGAGAGCGGCGTACGGGTGCGGAACACGT  57
            ||||||||||  ||||| | ||| || |  ||||||||||||||||||||||||||||||
Sbjct  40   GCCGAGCGGTAGAGATT-C-TTC-GGAATCTTGAGAGCGGCGTACGGGTGCGGAACACGT  96

Query  58   GTGCAACCTGCCTTTATCAGGGGGATAGCCTTTCGAAAGGAAGATTAATACCCCATAATA  117
            ||||||||||||||||||||||||||||||||||||||||||||||||||||||||||||
Sbjct  97   GTGCAACCTGCCTTTATCAGGGGGATAGCCTTTCGAAAGGAAGATTAATACCCCATAATA  156

Query  118  TATTATTCGGCATCGGGTGATATTGAAAACTACGGTGGATAGAGATGGGCACGCGCAAGA  177
            |||||   |||||| | |||||||||||||||||||||||||||||||||||||||||||
Sbjct  157  TATTAACTGGCATCAGTTGATATTGAAAACTACGGTGGATAGAGATGGGCACGCGCAAGA  216

Query  178  TTAGCTAGTTGGTGAGGTAACGGCTCACCAAGGCGACGATCTTTAGGGGGCCTGAGAGGG  237
            ||||||||||||||||||||||||||||||||||||||||||||||||||||||||||||
Sbjct  217  TTAGCTAGTTGGTGAGGTAACGGCTCACCAAGGCGACGATCTTTAGGGGGCCTGAGAGGG  276

Query  238  TGATCCCCCACACTGGTACTGAGACACGGACCAGACTCCTACGGGAGGCAGCAGTGAGGA  297
            ||||||||||||||||||||||||||||||||||||||||||||||||||||||||||||
Sbjct  277  TGATCCCCCACACTGGTACTGAGACACGGACCAGACTCCTACGGGAGGCAGCAGTGAGGA  336

Query  298  ATATTGGACAATGGGTGGAAGCCTGATCCAGCCATCCCGCGTGCAGGAAGACGGCCCTAT  357
            ||||||||||||||||||||||||||||||||||||||||||||||||||||||||||||
Sbjct  337  ATATTGGACAATGGGTGGAAGCCTGATCCAGCCATCCCGCGTGCAGGAAGACGGCCCTAT  396

Query  358  GGGTTGTAAACTGCTTTTATCTGGGGATAAACCTACTTACGTGTAAGTAGCTGAAGGTAC  417
            ||||||||||||||||||||||||||||||||||||||||||||||||||||||||||||
Sbjct  397  GGGTTGTAAACTGCTTTTATCTGGGGATAAACCTACTTACGTGTAAGTAGCTGAAGGTAC  456

Query  418  CAGAAGAATAAGCACCGGCTAACTCCGTGCCAGCAGCCGCGGTAATACGGAGGGTGCAAG  477
            ||||||||||||||||||||||||||||||||||||||||||||||||||||||||||||
Sbjct  457  CAGAAGAATAAGCACCGGCTAACTCCGTGCCAGCAGCCGCGGTAATACGGAGGGTGCAAG  516
```

```
Majority           GAGAGCGGCGTACGGGTGCGGAACACGTGTGCAACCTGCCTTTATCAGGGGGATAGCCTTTCGAAA
                            10        20        30        40        50        60
ATCC 13253 16S.seq GAGAGCGGCGTACGGGTGCGGAACACGTGTGCAACCTGCCTTTATCAGGGGGATAGCCTTTCGAAA
1.seq              GAGAGCGGCGTACGGGTGCGGAACACGTGTGCAACCTGCCTTTATCAGGGGGATAGCCTTTCGAAA
2.seq              GAGAGCGGCGTACGGGTGCGGAACACGTGTGCAACCTGCCTTTATCAGGGGGATAGCCTTTCGAAA
3.seq              GAGAGCGGCGTACGGGTGCGGAACACGTGTGCAACCTGCCTTTATCAGGGGGATAGCCTTTCGAAA
4.seq              GAGAGCGGCGTACGGGTGCGGAACACGTGTGCAACCTGCCTTTATCAGGGGGATAGCCTTTCGAAA
5.seq              GAGAGCGGCGTACGGGTGCGGAACACGTGTGCAACCTGCCTTTATCAGGGGGATAGCCTTTCGAAA
6.seq              GAGAGCGGCGTACGGGTGCGGAACACGTGTGCAACCTGCCTTTATCAGGGGGATAGCCTTTCGAAA
7.seq              GAGAGCGGCGTACGGGTGCGGAACACGTGTGCAACCTGCCTTTATCAGGGGGATAGCCTTTCGAAA
8.seq              GAGAGCGGCGTACGGGTGCGGAACACGTGTGCAACCTGCCTTTATCAGGGGGATAGCCTTTCGAAA

Majority           CCATAATATATTATTCGGCATCGGGTGATATTGAAAACTACGGTGGATAGAGATGGGCACGCGCAA
                            90        100       110       120       130       140
ATCC 13253 16S.seq CCATAATATATTAACTGGCATCAGTTGATATTGAAAACTACGGTGGATAGAGATGGGCACGCGCAA
1.seq              CCATAATATATTATTCGGCATCGGGTGATATTGAAAACTACGGTGGATAGAGATGGGCACGCGCAA
2.seq              CCATAATATATTATTCGGCATCGGGTGATATTGAAAACTACGGTGGATAGAGATGGGCACGCGCAA
3.seq              CCATAATATATTATTCGGCATCGGGTGATATTGAAAACTACGGTGGATAGAGATGGGCACGCGCAA
4.seq              CCATAATATATTATTCGGCATCGGGTGATATTGAAAACTACGGTGGATAGAGATGGGCACGCGCAA
5.seq              CCATAATATATTATTCGGCATCGGGTGATATTGAAAACTACGGTGGATAGAGATGGGCACGCGCAA
6.seq              CCATAATATATTATTCGGCATCGGGTGATATTGAAAACTACGGTGGATAGAGATGGGCACGCGCAA
7.seq              CCATAATATATTATTCGGCATCGGGTGATATTGAAAACTACGGTGGATAGAGATGGGCACGCGCAA
8.seq              CCATAATATATTATTCGGCATCGGGTGATATTGAAAACTACGGTGGATAGAGATGGGCACGCGCAA

Majority           TGAGGTAACGGCTCACCAAGGCGACGATCTTTAGGGGGCCTGAGAGGGTGATCCCCCACACTGGTA
                            170       180       190       200       210       220
ATCC 13253 16S.seq TGAGGTAACGGCTCACCAAGGCGACGATCTTTAGGGGGCCTGAGAGGGTGATCCCCCACACTGGTA
1.seq              TGAGGTAACGGCTCACCAAGGCGACGATCTTTAGGGGGCCTGAGAGGGTGATCCCCCACACTGGTA
2.seq              TGAGGTAACGGCTCACCAAGGCGACGATCTTTAGGGGGCCTGAGAGGGTGATCCCCCACACTGGTA
3.seq              TGAGGTAACGGCTCACCAAGGCGACGATCTTTAGGGGGCCTGAGAGGGTGATCCCCCACACTGGTA
4.seq              TGAGGTAACGGCTCACCAAGGCGACGATCTTTAGGGGGCCTGAGAGGGTGATCCCCCACACTGGTA
5.seq              TGAGGTAACGGCTCACCAAGGCGACGATCTTTAGGGGGCCTGAGAGGGTGATCCCCCACACTGGTA
6.seq              TGAGGTAACGGCTCACCAAGGCGACGATCTTTAGGGGGCCTGAGAGGGTGATCCCCCACACTGGTA
7.seq              TGAGGTAACGGCTCACCAAGGCGACGATCTTTAGGGGGCCTGAGAGGGTGATCCCCCACACTGGTA
8.seq              TGAGGTAACGGCTCACCAAGGCGACGATCTTTAGGGGGCCTGAGAGGGTGATCCCCCACACTGGTA

Majority           AGACTCCTACGGGAGGCAGCAGTGAGGAATATTGGACAATGGGTGGAAGCCTGATCCAGCCATCCC
                            250       260       270       280       290       300
ATCC 13253 16S.seq AGACTCCTACGGGAGGCAGCAGTGAGGAATATTGGACAATGGGTGGAAGCCTGATCCAGCCATCCC
1.seq              AGACTCCTACGGGAGGCAGCAGTGAGGAATATTGGACAATGGGTGGAAGCCTGATCCAGCCATCCC
2.seq              AGACTCCTACGGGAGGCAGCAGTGAGGAATATTGGACAATGGGTGGAAGCCTGATCCAGCCATCCC
3.seq              AGACTCCTACGGGAGGCAGCAGTGAGGAATATTGGACAATGGGTGGAAGCCTGATCCAGCCATCCC
4.seq              AGACTCCTACGGGAGGCAGCAGTGAGGAATATTGGACAATGGGTGGAAGCCTGATCCAGCCATCCC
5.seq              AGACTCCTACGGGAGGCAGCAGTGAGGAATATTGGACAATGGGTGGAAGCCTGATCCAGCCATCCC
6.seq              AGACTCCTACGGGAGGCAGCAGTGAGGAATATTGGACAATGGGTGGAAGCCTGATCCAGCCATCCC
7.seq              AGACTCCTACGGGAGGCAGCAGTGAGGAATATTGGACAATGGGTGGAAGCCTGATCCAGCCATCCC
8.seq              AGACTCCTACGGGAGGCAGCAGTGAGGAATATTGGACAATGGGTGGAAGCCTGATCCAGCCATCCC
```

```
Majority           GGCCCTATGGGTTGTAAACTGCTTTTATCTGGGGATAAACCTACTTACGTGTAAGTAGCTGAAGGT
                            330       340       350       360       370       380
ATCC 13253 16S.seq GGCCCTATGGGTTGTAAACTGCTTTTATCTGGGGATAAACCTACTTACGTGTAAGTAGCTGAAGGT
1.seq              GGCCCTATGGGTTGTAAACTGCTTTTATCTGGGGATAAACCTACTTACGTGTAAGTAGCTGAAGGT
2.seq              GGCCCTATGGGTTGTAAACTGCTTTTATCTGGGGATAAACCTACTTACGTGTAAGTAGCTGAAGGT
3.seq              GGCCCTATGGGTTGTAAACTGCTTTTATCTGGGGATAAACCTACTTACGTGTAAGTAGCTGAAGGT
4.seq              GGCCCTATGGGTTGTAAACTGCTTTTATCTGGGGATAAACCTACTTACGTGTAAGTAGCTGAAGGT
5.seq              GGCCCTATGGGTTGTAAACTGCTTTTATCTGGGGATAAACCTACTTACGTGTAAGTAGCTGAAGGT
6.seq              GGCCCTATGGGTTGTAAACTGCTTTTATCTGGGGATAAACCTACTTACGTGTAAGTAGCTGAAGGT
7.seq              GGCCCTATGGGTTGTAAACTGCTTTTATCTGGGGATAAACCTACTTACGTGTAAGTAGCTGAAGGT
8.seq              GGCCCTATGGGTTGTAAACTGCTTTTATCTGGGGATAAACCTACTTACGTGTAAGTAGCTGAAGGT

Majority           CACCGGCTAACTCCGTGCCAGCAGCCGCGGTAATACGGAGGGTGCAAGCGTTATCCGGATTTATTG
                            410       420       430       440       450       460
ATCC 13253 16S.seq CACCGGCTAACTCCGTGCCAGCAGCCGCGGTAATACGGAGGGTGCAAGCGTTATCCGGATTTATTG
1.seq              CACCGGCTAACTCCGTGCCAGCAGCCGCGGTAATACGGAGGGTGCAAGCGTTATCCGGATTTATTG
2.seq              CACCGGCTAACTCCGTGCCAGCAGCCGCGGTAATACGGAGGGTGCAAGCGTTATCCGGATTTATTG
3.seq              CACCGGCTAACTCCGTGCCAGCAGCCGCGGTAATACGGAGGGTGCAAGCGTTATCCGGATTTATTG
4.seq              CACCGGCTAACTCCGTGCCAGCAGCCGCGGTAATACGGAGGGTGCAAGCGTTATCCGGATTTATTG
5.seq              CACCGGCTAACTCCGTGCCAGCAGCCGCGGTAATACGGAGGGTGCAAGCGTTATCCGGATTTATTG
6.seq              CACCGGCTAACTCCGTGCCAGCAGCCGCGGTAATACGGAGGGTGCAAGCGTTATCCGGATTTATTG
7.seq              CACCGGCTAACTCCGTGCCAGCAGCCGCGGTAATACGGAGGGTGCAAGCGTTATCCGGATTTATTG
8.seq              CACCGGCTAACTCCGTGCCAGCAGCCGCGGTAATACGGAGGGTGCAAGCGTTATCCGGATTTATTG

Majority           GTAGGCGGACTGATAAGTCAGTGGTGAAATCCGACAGCTTAACTGTCGAACTGCCATTGATACTGT
                            490       500       510       520       530       540
ATCC 13253 16S.seq GTAGGCGGACTAGTAAGTCAGTGGTGAAATCCGACAGCTTAACTGTCGAACTGCCATTGATACTGC
1.seq              GTAGGCGGACTGATAAGTCAGTGGTGAAATCCGACAGCTTAACTGTCGAACTGCCATTGATACTGT
2.seq              GTAGGCGGACTGATAAGTCAGTGGTGAAATCCGACAGCTTAACTGTCGAACTGCCATTGATACTGT
3.seq              GTAGGCGGACTGATAAGTCAGTGGTGAAATCCGACAGCTTAACTGTCGAACTGCCATTGATACTGT
4.seq              GTAGGCGGACTGATAAGTCAGTGGTGAAATCCGACAGCTTAACTGTCGAACTGCCATTGATACTGT
5.seq              GTAGGCGGACTGATAAGTCAGTGGTGAAATCCGACAGCTTAACTGTCGAACTGCCATTGATACTGT
6.seq              GTAGGCGGACTGATAAGTCAGTGGTGAAATCCGACAGCTTAACTGTCGAACTGCCATTGATACTGT
7.seq              GTAGGCGGACTGATAAGTCAGTGGTGAAATCCGACAGCTTAACTGTCGAACTGCCATTGATACTGT
8.seq              GTAGGCGGACTGATAAGTCAGTGGTGAAATCCGACAGCTTAACTGTCGAACTGCCATTGATACTGT

Majority           GTTGAAGTGGCTGGAATAAGTAGTGTAGCGGTGAAATGCATAGATATTACTTAGAACACCAATTGC
                            570       580       590       600       610       620
ATCC 13253 16S.seq GTTGAAGTGGCTGGAATAAGTAGTGTAGCGGTGAAATGCATAGATATTACTTAGAACACCAATTGC
1.seq              GTTGAAGTGGCTGGAATAAGTAGTGTAGCGGTGAAATGCATAGATATTACTTAGAACACCAATTGC
2.seq              GTTGAAGTGGCTGGAATAAGTAGTGTAGCGGTGAAATGCATAGATATTACTTAGAACACCAATTGC
3.seq              GTTGAAGTGGCTGGAATAAGTAGTGTAGCGGTGAAATGCATAGATATTACTTAGAACACCAATTGC
4.seq              GTTGAAGTGGCTGGAATAAGTAGTGTAGCGGTGAAATGCATAGATATTACTTAGAACACCAATTGC
5.seq              GTTGAAGTGGCTGGAATAAGTAGTGTAGCGGTGAAATGCATAGATATTACTTAGAACACCAATTGC
6.seq              GTTGAAGTGGCTGGAATAAGTAGTGTAGCGGTGAAATGCATAGATATTACTTAGAACACCAATTGC
7.seq              GTTGAAGTGGCTGGAATAAGTAGTGTAGCGGTGAAATGCATAGATATTACTTAGAACACCAATTGC
8.seq              GTTGAAGTGGCTGGAATAAGTAGTGTAGCGGTGAAATGCATAGATATTACTTAGAACACCAATTGC
```

```
Majority           AAGTCTTAACTGACGCTGATGGACGAAAGCGTGGGGAGCGAACAGGATTAGATACCCTGGTAGTCC
                             650       660       670       680       690       700
ATCC 13253 16S.seq AAGTCTTAACTGACGCTGATGGACGAAAGCGTGGGGAGCGAACAGGATTAGATACCCTGGTAGTCC
1.seq              AAGTCTTAACTGACGCTGATGGACGAAAGCGTGGGGAGCGAACAGGATTAGATACCCTGGTAGTCC
2.seq              AAGTCTTAACTGACGCTGATGGACGAAAGCGTGGGGAGCGAACAGGATTAGATACCCTGGTAGTCC
3.seq              AAGTCTTAACTGACGCTGATGGACGAAAGCGTGGGGAGCGAACAGGATTAGATACCCTGGTAGTCC
4.seq              AAGTCTTAACTGACGCTGATGGACGAAAGCGTGGGGAGCGAACAGGATTAGATACCCTGGTAGTCC
5.seq              AAGTCTTAACTGACGCTGATGGACGAAAGCGTGGGGAGCGAACAGGATTAGATACCCTGGTAGTCC
6.seq              AAGTCTTAACTGACGCTGATGGACGAAAGCGTGGGGAGCGAACAGGATTAGATACCCTGGTAGTCC
7.seq              AAGTCTTAACTGACGCTGATGGACGAAAGCGTGGGGAGCGAACAGGATTAGATACCCTGGTAGTCC
8.seq              AAGTCTTAACTGACGCTGATGGACGAAAGCGTGGGGAGCGAACAGGATTAGATACCCTGGTAGTCC

Majority           GATTACTCGTTTTTGGGTTTAATGATTCAGAGACTAAGCGAAAGTGATAAGTAATCCACCTGGGGA
                             730       740       750       760       770       780
ATCC 13253 16S.seq GATTACTCGTTTTTGGGTTTTATGATTCAGAGACTAAGCGAAAGTGATAAGTAATCCACCTGGGGA
1.seq              GATTACTCGTTTTTGGGTTTAATGATTCAGAGACTAAGCGAAAGTGATAAGTAATCCACCTGGGGA
2.seq              GATTACTCGTTTTTGGGTTTAATGATTCAGAGACTAAGCGAAAGTGATAAGTAATCCACCTGGGGA
3.seq              GATTACTCGTTTTTGGGTTTAATGATTCAGAGACTAAGCGAAAGTGATAAGTAATCCACCTGGGGA
4.seq              GATTACTCGTTTTTGGGTTTAATGATTCAGAGACTAAGCGAAAGTGATAAGTAATCCACCTGGGGA
5.seq              GATTACTCGTTTTTGGGTTTAATGATTCAGAGACTAAGCGAAAGTGATAAGTAATCCACCTGGGGA
6.seq              GATTACTCGTTTTTGGGTTTAATGATTCAGAGACTAAGCGAAAGTGATAAGTAATCCACCTGGGGA
7.seq              GATTACTCGTTTTTGGGTTTTATGATTCAGAGACTAAGCGAAAGTGATAAGTAATCCACCTGGGGA
8.seq              GATTACTCGTTTTTGGGTTTTATGATTCAGAGACTAAGCGAAAGTGATAAGTAATCCACCTGGGGA

Majority           ATGAAACTCAAAGGAATTGACGGGGGCCCGCACAAGCGGTGGAGCATGTGGTTTAATTCGATGATA
                             810       820       830       840       850       860
ATCC 13253 16S.seq ATGAAACTCAAAGGAATTGACGGGGGCCCGCACAAGCGGTGGAGCATGTGGTTTAATTCGATGATA
1.seq              ATGAAACTCAAAGGAATTGACGGGGGCCCGCACAAGCGGTGGAGCATGTGGTTTAATTCGATGATA
2.seq              ATGAAACTCAAAGGAATTGACGGGGGCCCGCACAAGCGGTGGAGCATGTGGTTTAATTCGATGATA
3.seq              ATGAAACTCAAAGGAATTGACGGGGGCCCGCACAAGCGGTGGAGCATGTGGTTTAATTCGATGATA
4.seq              ATGAAACTCAAAGGAATTGACGGGGGCCCGCACAAGCGGTGGAGCATGTGGTTTAATTCGATGATA
5.seq              ATGAAACTCAAAGGAATTGACGGGGGCCCGCACAAGCGGTGGAGCATGTGGTTTAATTCGATGATA
6.seq              ATGAAACTCAAAGGAATTGACGGGGGCCCGCACAAGCGGTGGAGCATGTGGTTTAATTCGATGATA
7.seq              ATGAAACTCAAAGGAATTGACGGGGGCCCGCACAAGCGGTGGAGCATGTGGTTTAATTCGATGATA
8.seq              ATGAAACTCAAAGGAATTGACGGGGGCCCGCACAAGCGGTGGAGCATGTGGTTTAATTCGATGATA

Majority           CCAAGACTTAAATGGGAAATGACAGATTTAGAAATAGATCCTTCTTCGGACATTTTTCAAGGTGCT
                             890       900       910       920       930       940
ATCC 13253 16S.seq CCAAGACTTAAATGGGAAATGACAGATTTAGAAATAGATCCTTCTTCGGACATTTTTCAAGGTGCT
1.seq              CCAAGACTTAAATGGGAAATGACAGATTTAGAAATAGATCCTTCTTCGGACATTTTTCAAGGTGCT
2.seq              CCAAGACTTAAATGGGAAATGACAGATTTAGAAATAGATCCTTCTTCGGACATTTTTCAAGGTGCT
3.seq              CCAAGACTTAAATGGGAAATGACAGATTTAGAAATAGATCCTTCTTCGGACATTTTTCAAGGTGCT
4.seq              CCAAGACTTAAATGGGAAATGACAGATTTAGAAATAGATCCTTCTTCGGACATTTTTCAAGGTGCT
5.seq              CCAAGACTTAAATGGGAAATGACAGATTTAGAAATAGATCCTTCTTCGGACATTTTTCAAGGTGCT
6.seq              CCAAGACTTAAATGGGAATTGACAGACGCAGAAATGTGTTTTTCTTCGGACAATTTTCAAGGTGCT
7.seq              CCAAGACTTAAATGGGAAATGACAGATTTAGAAATAGATCCTTCTTCGGACATTTTTCAAGGTGCT
8.seq              CCAAGACTTAAATGGGAATTGACAGATTTAGAAATAGATTTTTCTTCGGACATTTTTCAAGGTGCT
```

```
Majority           AGCTCGTGCCGTGAGGTGTTAGGTTAAGTCCTGCAACGAGCGCAACCCCTGTCACTAGTTGCTAAC

                            970       980       990      1000      1010      1020

ATCC 13253 16S.seq AGCTCGTGCCGTGAGGTGTTAGGTTAAGTCCTGCAACGAGCGCAACCCCTGTCACTAGTTGCTAAC
1.seq              AGCTCGTGCCGTGAGGTGTTAGGTTAAGTCCTGCAACGAGCGCAACCCCTGTCACTAGTTGCTAAC
2.seq              AGCTCGTGCCGTGAGGTGTTAGGTTAAGTCCTGCAACGAGCGCAACCCCTGTCACTAGTTGCTAAC
3.seq              AGCTCGTGCCGTGAGGTGTTAGGTTAAGTCCTGCAACGAGCGCAACCCCTGTCACTAGTTGCTAAC
4.seq              AGCTCGTGCCGTGAGGTGTTAGGTTAAGTCCTGCAACGAGCGCAACCCCTGTCACTAGTTGCTAAC
5.seq              AGCTCGTGCCGTGAGGTGTTAGGTTAAGTCCTGCAACGAGCGCAACCCCTGTCACTAGTTGCTAAC
6.seq              AGCTCGTGCCGTGAGGTGTTAGGTTAAGTCCTGCAACGAGCGCAACCCCTGTCACTAGTTGCTAAC
7.seq              AGCTCGTGCCGTGAGGTGTTAGGTTAAGTCCTGCAACGAGCGCAACCCCTGTCACTAGTTGCTAAC
8.seq              AGCTCGTGCCGTGAGGTGTTAGGTTAAGTCCTGCAACGAGCGCAACCCCTGTCACTAGTTGCTAAC

Majority           TCTAGTGAGACTGCCTACGCAAGTAGAGAGGAAGGTGGGGATGACGTCAAATCATCACGGCCCTTA

                           1050      1060      1070      1080      1090      1100

ATCC 13253 16S.seq TCTAGTGAGACTGCCTACGCAAGTAGAGAGGAAGGTGGGGATGACGTCAAATCATCACGGCCCTTA
1.seq              TCTAGTGAGACTGCCTACGCAAGTAGAGAGGAAGGTGGGGATGACGTCAAATCATCACGGCCCTTA
2.seq              TCTAGTGAGACTGCCTACGCAAGTAGAGAGGAAGGTGGGGATGACGTCAAATCATCACGGCCCTTA
3.seq              TCTAGTGAGACTGCCTACGCAAGTAGAGAGGAAGGTGGGGATGACGTCAAATCATCACGGCCCTTA
4.seq              TCTAGTGAGACTGCCTACGCAAGTAGAGAGGAAGGTGGGGATGACGTCAAATCATCACGGCCCTTA
5.seq              TCTAGTGAGACTGCCTACGCAAGTAGAGAGGAAGGTGGGGATGACGTCAAATCATCACGGCCCTTA
6.seq              TCTAGTGAGACTGCCTACGCAAGTAGAGAGGAAGGTGGGGATGACGTCAAATCATCACGGCCCTTA
7.seq              TCTAGTGAGACTGCCTACGCAAGTAGAGAGGAAGGTGGGGATGACGTCAAATCATCACGGCCCTTA
8.seq              TCTAGTGAGACTGCCTACGCAAGTAGAGAGGAAGGTGGGGATGACGTCAAATCATCACGGCCCTTA

Majority           CACGTGCTACAATGGCCGGTACAGAGGGCAGCTACCTAGTGATAGGATGCAAATCTCGAAAGCCGG

                           1130      1140      1150      1160      1170      1180

ATCC 13253 16S.seq CACGTGCTACAATGGCCGGTACAGAGGGCAGCTACCTAGCGATAGGATGCAAATCTCGAAAGCCGG
1.seq              CACGTGCTACAATGGCCGGTACAGAGGGCAGCTACCTAGTGATAGGATGCAAATCTCGAAAGCCGG
2.seq              CACGTGCTACAATGGCCGGTACAGAGGGCAGCTACCTAGTGATAGGATGCAAATCTCGAAAGCCGG
3.seq              CACGTGCTACAATGGCCGGTACAGAGGGCAGCTACCTAGTGATAGGATGCAAATCTCGAAAGCCGG
4.seq              CACGTGCTACAATGGCCGGTACAGAGGGCAGCTACCTAGTGATAGGATGCAAATCTCGAAAGCCGG
5.seq              CACGTGCTACAATGGCCGGTACAGAGGGCAGCTACCTAGTGATAGGATGCAAATCTCGAAAGCCGG
6.seq              CACGTGCTACAATGGCCGGTACAGAGGGCAGCTACCTAGTGATAGGATGCAAATCTCGAAAGCCGG
7.seq              CACGTGCTACAATGGCCGGTACAGAGGGCAGCTACCTAGTGATAGGATGCAAATCTCGAAAGCCGG
8.seq              CACGTGCTACAATGGCCGGTACAGAGGGCAGCTACCTAGTGATAGGATGCAAATCTCGAAAGCCGG

Majority           GGAGTCTGCAACTCGACTCTATGAAGCTGGAATCGCTAGTAATCGCGCATCAGCCATGGCGCGGTG

                           1210      1220      1230      1240      1250      1260

ATCC 13253 16S.seq GGAGTCTGCAACTCGACTCTATGAAGCTGGAATCGCTAGTAATCGCGCATCAGCCATGGCGCGGTG
1.seq              GGAGTCTGCAACTCGACTCTATGAAGCTGGAATCGCTAGTAATCGCGCATCAGCCATGGCGCGGTG
2.seq              GGAGTCTGCAACTCGACTCTATGAAGCTGGAATCGCTAGTAATCGCGCATCAGCCATGGCGCGGTG
3.seq              GGAGTCTGCAACTCGACTCTATGAAGCTGGAATCGCTAGTAATCGCGCATCAGCCATGGCGCGGTG
4.seq              GGAGTCTGCAACTCGACTCTATGAAGCTGGAATCGCTAGTAATCGCGCATCAGCCATGGCGCGGTG
5.seq              GGAGTCTGCAACTCGACTCTATGAAGCTGGAATCGCTAGTAATCGCGCATCAGCCATGGCGCGGTG
6.seq              GGAGTCTGCAACTCGACTCTATGAAGCTGGAATCGCTAGTAATCGCGCATCAGCCATGGCGCGGTG
7.seq              GGAGTCTGCAACTCGACTCTATGAAGCTGGAATCGCTAGTAATCGCGCATCAGCCATGGCGCGGTG
8.seq              GGAGTCTGCAACTCGACTCTATGAAGCTGGAATCGCTAGTAATCGCGCATCAGCCATGGCGCGGTG
```

```
Majority              CCTTGTACACACCGCCCGTCAAGCCATGGAAGCTGGGGGTACCTGAAGTCGGTGACCGTAAAAGGA

                                1290      1300      1310      1320      1330      1340

ATCC 13253 16S.seq    CCTTGTACACACCGCCCGTCAAGCCATGGAAGCTGGGGGTACCTGAAGTCGGTGACCGTAAAAGGA
1.seq                 CCTTGTACACACCGCCCGTCAAGCCATGGAAGCTGGGGGTACCTGAAGTCGGTGACCGTAAAAGGA
2.seq                 CCTTGTACACACCGCCCGTCAAGCCATGGAAGCTGGGGGTACCTGAAGTCGGTGACCGTAAAAGGA
3.seq                 CCTTGTACACACCGCCCGTCAAGCCATGGAAGCTGGGGGTACCTGAAGTCGGTGACCGTAAAAGGA
4.seq                 CCTTGTACACACCGCCCGTCAAGCCATGGAAGCTGGGGGTACCTGAAGTCGGTGACCGTAAAAGGA
5.seq                 CCTTGTACACACCGCCCGTCAAGCCATGGAAGCTGGGGGTACCTGAAGTCGGTGACCGTAAAAGGA
6.seq                 CCTTGTACACACCGCCCGTCAAGCCATGGAAGCTGGGGGTACCTGAAGTCGGTGACCGTAAAAGGA
7.seq                 CCTTGTACACACCGCCCGTCAAGCCATGGAAGCTGGGGGTACCTGAAGTCGGTGACCGTAAAAGGA
8.seq                 CCTTGTACACACCGCCCGTCAAGCCATGGAAGCTGGGGGTACCTGAAGTCGGTGACCGTAAAAGGA
```
